# The predictive utility of circulating PCSK9 levels on diabetes mellitus

**DOI:** 10.1186/s12933-021-01226-5

**Published:** 2021-02-13

**Authors:** Jia Peng, Cheng-Gang Zhu, Jian-Jun Li

**Affiliations:** State Key Laboratory of Cardiovascular Diseases, FuWai Hospital, National Center for Cardiovascular Diseases, Chinese Academy of Medical Sciences and Peking Union Medical College, No 167 BeiLiShi Road, XiCheng District, Beijing, 100037 China

**Keywords:** Proprotein convertase subtilisin/kexin type 9, Type 2 diabetes mellitus, Risks

## Abstract

Increasing data including ours have suggested that proprotein convertase subtilisin/kexin type 9 (PCSK9), a novel regulator of cholesterol metabolism, may also play an important role in the development of type 2 diabetes mellitus (T2DM) and is associated with clinical outcomes in diabetic patients. Previous studies revealed that elevated plasma PCSK9 levels had a higher incidence of new-onset T2DM. Moreover, the results of available epidemiological, preclinical, and clinical studies have indicated that plasma PCSK9 concentration is correlated with glycemic parameters and can predict the adverse cardiovascular events in diabetic patients with coronary artery disease. However, there is currently no general agreement about the association of PCSK9 with T2DM. The usefulness of the circulating PCSK9 concentration as a predictor for the risk of new-onset T2DM should be clinically prudential.

We have read the article with great interest by Shi et al. on the relationship of circulating proprotein convertase subtilisin/kexin type 9 (PCSK9) concentration with the risk of incident type 2 diabetes mellitus (T2DM) in prediabetic Chinese population [1]. They concluded that elevated PCSK9 levels were associated with an increased incidence of T2DM in female individuals with prediabetes (pre-DM).

In fact, the best-known function of PCSK9 is to take part in cholesterol metabolism by acting on low-density lipoprotein receptor expressed in the liver, resulting in a remark increase of circulating low-density lipoprotein cholesterol [2]. Additionally, circulating PCSK9 concentration has also been proposed to be a novel biomarker for predicting the coronary artery calcification, coronary severity and worse cardiovascular outcomes in different clinical settings including general population, subjects with stable coronary artery disease (CAD), patients with familial hypercholesterolemia, and individuals with arterial fibrillation [2]. Besides, the association between PCSK9 and T2DM has becoming an interesting issue since its discovery. It was previously showed that plasma PCSK9 levels were higher in patients with T2DM [3]. Moreover, the results available from epidemiology, preclinical and clinical studies suggested a positive correlation of circulating PCSK9 concentration with glycemic parameters and risks of T2DM [3]. Meanwhile, a recent animal study has revealed that downregulating PCSK9 can ameliorate lipid and glucose metabolism [4].

Clinically, pre-DM, the intermediate hyperglycemia, is a high-risk state for developing T2DM. Emerging data have already supported that disturbed glycemic metabolism plays a major role in atherosclerosis and cardiovascular diseases. Hence, it is critical to provide methods for identification of individuals at risk of pre-DM or T2DM on the basis of indices available to doctors [5]. Additionally, understanding the progression of pre-DM, identifying influencing factors for T2DM in a timely and accurate manner and making interventions to reduce the risk for T2DM are necessary among the patients with pre-DM. It has indicated that several factors are associated with the risks of T2DM among prediabetic subjects, such as ageing, body mass index, obesity, family history of T2DM, elevated glycemic parameters, fasting insulin, and albuminuria [5].

Interestingly, Shi et al. found a positive relationship with elevated baseline circulating PCSK9 levels and incidence of T2DM in female population with pre-DM during 3.1 years follow-up, which was the first study to undertake a longitudinal analysis of circulating PCSK9 concentration and the development of T2DM in prediabetic individuals [1]. Although their finding provided an additional information on the association with circulating PCSK9 concentration and the incident risk of new-onset T2DM, there were several issues might be worth deliberating before making a conclusion.

First of all, a gender imbalance of patients enrolled in this study might be a problem. Numerous studies have indicated that a gender difference can be found, which leads to different outcomes, for example, men have higher risks of CAD compared with women [6]. The current study also found a remark gender difference in the association of PCSK9 levels with incidence of T2DM in women but not men. As we have already known that men appeared to be at greater risk of T2DM at a younger age compared to women [7]. What’s more, in this study the percentage of females had an absolute advantage (67 %, n = 2817) than that of males (33.0 %, n = 1388), which might influence the results. If the numbers of males in the present study was analogous, the correlation of PCSK9 concentration might also be similar. Thereby, further investigation may be needed in young, well-gender balanced populations. Alternatively, an age- and sex-matched case–control study would be suggested.

Secondly, previous studies showed that circulating PCSK9 levels could be affected by multiple factors, including exercise, smoking, alcohol or tea consumption, and lipid-lowering drugs [8]. As shown by prior observations, several lipid-lowering medications could significantly up-regulate the expression of PCSK9 genes and increase circulating PCSK9 concentration even if a single dose of statin, ezetimibe, Xuezhikang, or berberine was administrated [8]. Notably, the duration of lipid-lowering drug treatment is also an important factor for affecting PCSK9 levels. In order to avoid this impact on the baseline levels of PCSK9, it is recommended to exclude patients who took any lipid-lowering drugs within 3 months before enrollment. In this article, authors did not provide detailed information regarding to tea consumption and the usage of lipid-lowering agents. In addition, for prediabetic patients, lifestyle intervention is the cornerstone of T2DM prevention, with evidence of a 40–70% relative-risk reduction during follow-up time [5]. Likewise, most guidelines have recommended the high-intensity life intervention on preferred T2DM prevention intervention [9]. Nevertheless, Shi et al. only recorded physical activity of each prediabetic individual at baseline but no information concerning their lifestyle intervention including diet and exercise during the follow-up time. Despite the sample size of the current study was relatively large, the lack of these important baseline and follow-up information might weaken the conclusion on the relationship of PCSK9 with incidence of T2DM. A prospective, randomized case–control study may be needed to make sure that PCSK9 is associated with increased risk of new-onset T2DM in patients with pre-DM.

Thirdly, it is also worth noting that the individuals with pre-DM is considered to have higher risk of developing T2DM compared to subjects with normal glucose regulation (NGR) [5]. Furthermore, in analysis of our data published recently [3], all 1225 patients with stable CAD were further divided into three subgroups according to diabetic status (T2DM: n = 377, pre-DM: n = 489, NGR: n = 359) and an ascending increment of baseline levels of PCSK9 were observed [T2DM vs. pre-DM vs. NGR: 246.51 (199.43, 292.22) vs. 238.88 (198.53, 275.65) vs. 225.31 (185.95, 263.36) ng/mL, Fig. 1]. Hence, it might be much better that a subgroup of NGR was covered in their study to examine the relation of circulating PCSK9 concentration with future diabetic incidence. Generally, the predictive utility of circulating PCSK9 levels on T2DM need to be further confirmed.

**Fig. 1:**
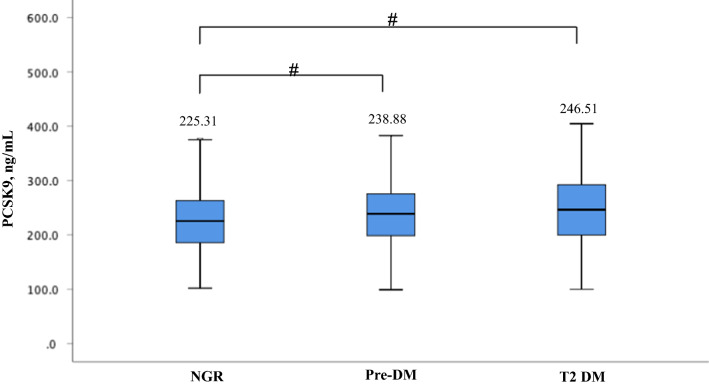
Circulating PCSK9 levels according to diabetic status. *PCSK9* proprotein convertase subtilisin/kexin type 9, *NGR* normal glucose regulation, *Pre-DM* prediabetes, *T2DM* type 2 diabetes mellitus. ^#^ *p* < 0.01

## Data Availability

Not applicable.

## Abbreviations

PCSK9: Proprotein convertase subtilisin/kexin type 9
T2DM: Type 2 diabetes mellitus
pre-DM: Prediabetes
CAD: Coronary artery disease
NGR: Normal glucose regulation
